# An improved microscale method for extraction of phenolic acids from maize

**DOI:** 10.1186/s13007-017-0235-x

**Published:** 2017-10-10

**Authors:** Mariana Zavala-López, Silverio García-Lara

**Affiliations:** Biotechnology Center, School of Engineering and Science, Tecnologico de Monterrey, Campus Monterrey, Eugenio Garza Sada 2501, C.P. 64849 Monterrey, N.L. Mexico

**Keywords:** Extraction, Phenolic acid, Maize, Microscale

## Abstract

**Background:**

Phenolic acids are a major group of secondary metabolites widely distributed in plants. In the case of maize, the major proportion of these metabolites occurs in the edible grain and their antioxidant activities are associated with improvements in human health. However, conventional extraction of secondary metabolites is very time consuming and generates a substantial amount of solvent waste. One approach to resolve these limitations is the use of microscale approaches, which minimize the quantity of solvents required, as well as the sample amounts and processing times. The objective of this work was to develop an improved microscale method for extraction of phenolic acids from maize and to compare it with a conventional extraction method.

**Results:**

The improved microscale extraction method, coupled with an HPLC–DAD detection method, allowed identification of ferulic acid, *p*-coumaric acid in its free and bound form, and some diferulic acids. In its free form, *p*-coumaric acid ranged in content from 2.4 to 6.5 µg/g dry weight (dw) using the conventional method and 7.7 to 54.8 µg/g dw using the improved microscale method. Free ferulic acid content ranged from 2.6 to 12.9 µg/g dw for the conventional method and 16.8 to 181.7 µg/g dw for the improved microscale method. In its bound form, *p*-coumaric acid ranged in content from 6.0 to 30.6 µg/g dw for the traditional method and 34.4 to 138.6 µg/g dw for the improved microscale method. Bound ferulic acid ranged from 131.8 to 427.5 µg/g dw for the conventional method and 673.8 to 1702.7 µg/g dw for the improved microscale method. The coefficient of variation associated was lower for the improved microscale method than for the conventional method, thereby assuring the replicability of the process.

**Conclusions:**

The improved microscale method proposed here increases the extraction power and batch capacity, while reducing the sample quantity, solvent amounts and extraction time. It also achieves a better replicability with a lower coefficient of variation than is possible with conventional extraction.

**Electronic supplementary material:**

The online version of this article (doi:10.1186/s13007-017-0235-x) contains supplementary material, which is available to authorized users.

## Background

Phenolic compounds are a major group of secondary metabolites widely distributed in plants. The phenolics found in maize have drawn attention because of their associated benefits for human health. These health effects are mainly associated with the antioxidant activity of these compounds that aids in protection of the plant itself against pests [1]. The phenolic profile varies according to the matrix analyzed: in the case of maize, the major proportion of phenolics are bound to the cell wall, and the two most prominent phenolic compounds are the phenolic acids, *trans*-ferulic acid and *p*-coumaric acid [2].

Analysis of these metabolites requires an efficient extraction procedure that provides a high extraction rate as well as a high specificity for the compounds of interest. Conventional extraction procedures involve the use of different solvents that take into consideration the polarity of the compounds to be extracted [3]. A typical procedure can be divided into two major steps: free phenolic extraction and bound phenolic extraction. The first step consists of an alcohol extraction to obtain the soluble compounds, while the second step is an alkali hydrolysis to free the bound phenolics from the cell wall; this is followed by a series of several washes to purify the sample [4]. One of the major drawbacks of this procedure is its requirement for large quantities of different solvents (i.e., methanol, hexane and ethyl acetate), which generates a significant quantity of toxic solvent waste [3]. The procedure is also very time consuming, making the handling of several samples at once a challenging task.

Alternative methods for phenolic acid extraction have been developed, mainly using techniques of microwave-assisted extraction [5], pulsed-electric field [6], ultrasound, and supercritical fluid extraction [3]. However, these techniques still present other major disadvantages, such as requirements for further purification processes [7], lack of identification power [8] and lower recovery yield [9], or they are still under development for their use in maize. For these reasons, microscale approaches have gained attention in recent years, as they have the potential to resolve the major issues of conventional extractions by minimizing the quantity of solvents and sample required while shortening the processing time. These adaptations allow the handling of larger sets of samples per batch in a shorter amount of time [10–12]. The objective of the present work was to develop an improved microscale extraction method for phenolic acids in maize and to compare its efficacy to that of a conventional macroscale extraction method.

## Methods

### Materials

A group of nineteen temperate yellow hybrids maize materials was used for the evaluation of the conventional and microscale phenolic acid extraction procedures. All maize hybrids were harvested during 2014 and kindly donated by a Local private company. The ears were dried, shelled, milled, and stored at 4 °C until further processing. A list of materials, along with proximal analysis, can be found in Additional file 1. Maize kernels were ground to a fine powder (< 1 mm) using two grinding steps (Krups GX4100, MX, followed by Retsch MM400) and passed through a sieve (US. 60). The ground maize material was stored at − 20 °C.

### Reagents

NaOH and Folin–Ciocalteu reagents were purchased from Macron, CH. 2 M HCl was obtained from J.T. Baker, MX. N-hexane was purchased at DEQ, MX. Ethyl acetate was obtained from Burdick & Jackson, Muskegon, MI. All HPLC solvents and alcohols were obtained from BDH, West Chester, PA. HPLC grade (> 99%) standards for gallic acid, trifluoroacetic acid, p-coumaric acid and ferulic acid were purchased from Sigma-Aldrich, St. Louis, MO.

### Conventional phenolic acid extraction

The conventional phenolic acid extraction methodology used was a modified method of Adom & Liu [2]. Briefly, 10 mL of 80% ethanol were added to 1 g of ground maize sample. The samples were incubated at 25 °C for 15 min with constant agitation at 500 rpm (Vortemp 1550, Labnet, Woodbridge, NJ) and then centrifuged (VWR 1814, USA) for 10 min at 10,000 rpm and 4 °C. The supernatant was decanted and stored at − 20 °C until analysis. The residue pellet was used for extraction of bound phenolics, as follows. Ten mL of 2 M NaOH were added to the residue pellet. The resuspended pellet was mixed at 3000 rpm on a vortex mixer (Vortex VWR, USA). An alkaline hydrolysis treatment was carried out by incubation at 90 °C for 1.5 h and constant agitation at 500 rpm. The samples were then acidified with 14 mL of 2 M HCl and the pH of the samples was verified as being between pH 2 and 3 using pH strips (Macherey–Nagel, DK). Lipids were removed by adding 12 mL of n-hexane to the samples, followed by vortexing for 5 min at 2500 rpm, incubation at 25 °C for 10 min with constant agitation at 500 rpm, and centrifugation for 10 min at 10,000 rpm. The upper hexane phase in the resulting three-layered system was discarded and this procedure was repeated twice more, for a total of three n-hexane washes. Ten mL of ethyl acetate were then added to recover the phenolic acids. The samples were mixed at 3000 rpm, incubated at 25 °C for 10 min with constant agitation at 500 rpm and then centrifuged again for 10 min at 10,000 rpm. The upper ethyl acetate phase was recovered from the resulting three-layer system that formed. The ethyl acetate was removed by evaporating to dryness in an extraction hood and the sample was resuspended in 5 mL of 50% methanol. This procedure was repeated to complete five ethyl acetate washes. The extracts were stored at − 20 °C until analysis.

### Improved microscale phenolic acid extraction

The improvement in phenolic acid extraction, achieved through the use of a microscale process, was performed as follows. Instead of ethanol, 80% methanol was used as the extraction solvent for soluble phenolic acids; 0.7 mL were added to 50 mg of maize sample and mixed for 5 min at 2500 rpm on a vortex (Vortex VWR, USA). The sample was incubated at 25 °C for 15 min at a constant agitation of 500 rpm (Vortemp 1550, Labnet, Woodbridge, NJ), followed by centrifugation (VWR 1814, USA) for 10 min at 5000 rpm. The supernatant was decanted and stored at − 20 °C until analysis. The bound phenolics were extracted from the pellet residue formed in the previous step using a microscale procedure. First, the volume of 2 M NaOH added to the pellet was reduced from 10 to 0.5 mL. The samples were flushed with nitrogen gas to protect them from oxygen degradation and vortexed for 5 min at 2500 rpm.

The alkaline hydrolysis was conducted at a temperature maintained at 90 °C and a constant agitation at 500 rpm, but the procedure time was reduced to 1 h (compared to 1.5 h). After hydrolysis, the sample was acidified with 0.5 mL of 2 M HCl, and a value of pH 2 was verified with pH strips; otherwise the pH was adjusted with the appropriate amount of 2 M HCl. Lipid removal was achieved by adding only 0.8 mL of n-hexane to the samples, which were then vortexed for 5 min at 2500 rpm, incubated at 25 °C for 10 min with a constant agitation of 500 rpm, and centrifuged for 10 min at 5000 rpm. The hexane layer from the resulting three-layered system was discarded (upper phase). This procedure was repeated in order to complete two n-hexane washes. The bound phenolic acids were recovered by adding 0.8 mL of ethyl acetate, mixing the sample for 5 min at 2500 rpm, incubating at 25 °C for 10 min with a constant agitation of 500 rpm and centrifuged for 10 min at 5000 rpm. The ethyl acetate layer (upper phase) from the three-layered system formed was recovered. The number of repetitions of this process was reduced to only three complete ethyl acetate washes. Ethyl acetate was evaporated to dryness in an extraction hood and the sample was resuspended in 200 µL of 50% methanol, filtered through a 0.45 µm GHP and Nylon filter (Pall Life Sciences, Ann Harbor, MI), and stored at − 20 °C until further analysis.

### Determination of total free and bound phenolic acids

The total free and bound phenolics extracted using the improved and conventional extraction methods were quantified using the Folin–Ciocalteu assay according to Urias-Peraldí et al. [13]. Briefly, 100 µL of 10% folin reagent were added to 20 µL of sample in a 96-well microplate. After 5 min, the reaction was neutralized with 80 µL of Na_2_CO_3_ (7.5% w/v). Incubation was performed for 2 h at room temperature. Total phenolic acids were quantified using a microplate reader (Synergy ™ HT Multi-Detection, BioTek, Inc., Winooski, VT) at 765 nm. Gallic acid was used as a standard and total phenolic content was expressed as μg of gallic acid equivalents per 100 mg dry weight (GAE/100 mg dw).

### Determination of free and bound phenolic acids by HPLC

Extracted phenolic acids were analyzed according to the method of Ayala-Soto et al. [14] using an HPLC (Agilent 1100 Santa Clara, CA) coupled with a photodiode array (PDA) detector (Agilent G1315D, Santa Clara, CA). Linear gradient elution was performed with HPLC-grade water (acidified to pH 2 with trifluoroacetic acid) and acetonitrile, at a flow rate of 0.6 mL/min at 25 °C. Phenolic acids were separated on a Zorbax SB-Aq, 4.6 mm ID × 150 mm (3.5 µm) reverse phase column. The Chemstation software (for LC; Copyright© Agilent Technologies, 1990–2003) was used to process the data and command the equipment. Peak identification of *trans*-ferulic acid and *p*-coumaric acid was based on the retention times of known standards. Diferulic acids were identified according to the retention times reported previously [14–16] and were reported as equivalents of ferulic acid.

### Statistical analysis

Phenolic acid extractions were done in triplicate and total randomization of replicates was used to minimize the bias of the assay. Statistix ^®^ v.8 was used for the statistical analysis of the data. Statistical analysis included analysis of variance (ANOVA, α = 0.05%) between methods of extraction and between samples. Significant differences between the means of samples were further analyzed by least significant difference (LSD, α = 0.05). Box plot diagrams were used to show the dispersion within each method of extraction for the different maize samples and to compare between methods, as well as to reveal the dispersion in the coefficient of variation associated to each extraction method. A p value < 0.05 was considered statistically significant.

## Results

The new microscale method for phenolic acid extraction was validated in tandem with the conventional method by extracting 19 maize samples by both the conventional and the improved methods. Table 1 summarizes the principal differences between both methodologies, considering the amount of sample, procedure time, required solvents and generated solvent waste. The required sample was reduced from 1000 to 50 mg with the microscale method; this represents a reduction of 95% of the required sample. Drying time was decreased over 70%, solvent consumption for the bound phenolic extraction was lowered by 65% and solvent waste was reduced by in 67%. The microscale protocol also increased the number of samples that an operator was able to process per batch. Sixty-six samples were handled in a single batch, compared with the 9 samples handled by the conventional method, representing an increase of 633%. Comparison of the conventional method with the microscale method proposed here revealed several advantages of the improved method, especially when sample quantity was a constraint. The improved method allowed for a reduction in the quantity of sample and solvents required, extraction time, and solvent waste generated, while increasing the number of samples analyzed per batch.

**Table 1:** Comparison of the main steps of the process by conventional and improved methods

Step	Conventional method	Improved method	Δ* (%)
Required sample (mg)	1000	50	95
Extraction time			
Free phenolics (min)	15	30	− 100
Bound phenolics (h)	12	10	17
Drying time (days)	7	2	71
Consumed solvents			
Free phenolics (mL)	90	46	49
Bound phenolics (mL)	1080	376	65
Solvents waste (mL)	324	106	67
Number of samples per batch^a^	9	66	− 633

Δ*: Difference between conventional and improved methods expressed as percentages

^a^According to the capabilities of the available centrifuge equipment

**Fig. 1:**
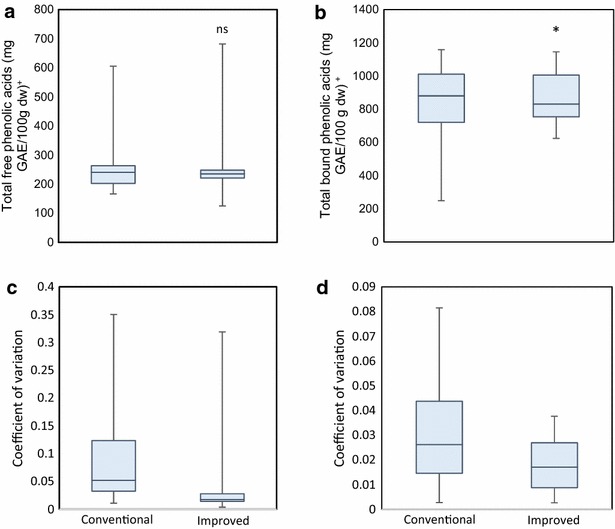
Box plot comparison of total phenolic acids in maize seeds. **a** Total free phenolic acids. **b** Total bound phenolic acids. **c** Coefficient of variation of total free phenolic acid quantification; **d** coefficient of variation of total bound phenolic acids. Horizontal line represents the median of total values. Vertical line represents the maximum and minimum value. Box covers the 75 and 25 percentiles. [^+^Results are expressed as mg of gallic acid equivalent per 100 g of dry weight (mg GAE/100 g dw). Significant differences between the methods were established by ANOVA (α = 0.05), *ns* no significance, * p < 0.05; ** p < 0.001; *** p < 0.001]

Total phenolic acids were quantified in free and bound forms by the conventional and improved methods. Figure 1 shows a similar rate of extraction for total phenolic acids in free form by both extraction methodologies. In the free form, phenolic acids extracted by the conventional method ranged from 166.4 to 605.3 mg GAE/100 g dw while the microscale method achieved a similar extraction range that fluctuated from 125.2 to 682.0 mg GAE/100 g dw; this difference was not statistically significant (Additional file 2). Comparison of the coefficient of variations for the two methodologies showed that the improved protocol had a reduced range, where most of the data fell below 0.05, whereas the conventional method had a higher range, where most of the data rested between 0.12 and 0.03.

Both methodologies were significantly different in terms of the phenolic concentration determined in the bound form (Fig. 1). Bound phenolic acids extracted by the conventional method ranged from 248.3 to 1158.4 mg GAE/100 g dw, whereas the improved method achieved a higher extraction that ranged from 623.9 to 1165.3 mg GAE/100 g dw (Additional file 3). The lowest coefficient of variation for both methodologies was under 0.01; however, the highest value with the conventional method reached 0.08, whereas the improved method did not exceed 0.04. The improved method achieved an extraction of total phenolic acids that was as efficient as the conventional method, and superior to the conventional method for bound phenolic acids, with a lower variation in the amounts of both total free and bound phenolic acids.

**Fig. 2:**
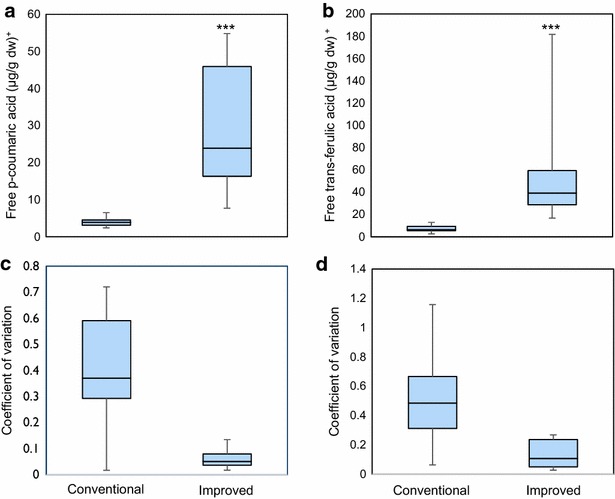
Box plot comparison of free phenolic acids extracted from maize seeds by the conventional and improved methods. **a** Determination of *p*-coumaric acid. **b** Determination of *trans*-ferulic acid. **c** Standard deviation for *p*-coumaric acid. **d** Standard deviation for ferulic acid. Horizontal line represents the median. Vertical line represents the maximum and minimum value. Box covers the 75th and 25th percentiles. [^+^Results are expressed as µg per g dry weight (µg/g dw). Significant differences between the methods were established by ANOVA (α = 0.05), *ns* no significance, * p < 0.05; ** p < 0.001; *** p < 0.001]

The results for free phenolic acids, quantified individually by HPLC, are shown in Fig. 2. Significant differences were found between the extraction methods for free *p*-coumaric and *trans*-ferulic acid. In the free form, the microscale method ranged from 7.7 to 54.8 µg/g dw for *p*-coumaric acid and 16.8 to 181.7 µg/g dw for *trans*-ferulic acid, respectively (Additional file 3). Thus, the improved method increased the extraction by over sevenfold for *p*-coumaric acid and eightfold for *trans*-ferulic acid. The improved method showed an important lower variation when compared with the conventional method, as the coefficient of variation was lower than 0.15 when compared with the coefficient of variation of over 0.3 observed with the conventional method. The conventional method also showed a higher range of variation from nearly 0 to over 0.8. Thus, the improved microscale method achieved a higher extraction for both *p*-coumaric and *trans*-ferulic acids in their free form, with a reduced variation, when compared to the conventional method.

**Fig. 3:**
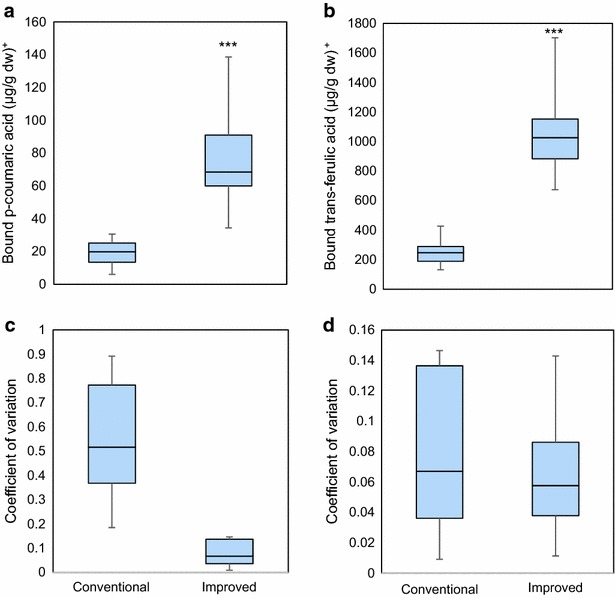
Box plot comparison of bound phenolic acids in maize seeds extracted by the conventional and improved methods. **a** Determination of *p*-coumaric acid. **b** Determination of *trans*-ferulic acid. **c** Standard deviation of *p*-coumaric acid. **d** Standard deviation ferulic acid. Horizontal line represents the median. Vertical line represents the maximum and minimum value. Box covers the 75th and 25th percentiles. [^+^Results expressed as µg per g dry weight (µg/g dw). Significant differences between the methods were established by ANOVA (α = 0.05), ns: no significance, * p < 0.05; ** p < 0.001; *** p < 0.001]

Individual bound phenolic acids extracted by the conventional and improved methods and quantified by HPLC are shown in Fig. 3. Significant differences were found between the extraction methods for both the bound phenolic acids quantified. The microscale method achieved a greater extraction of *p*-coumaric acid, ranging from 34.4 to 138.6 µg/g dw, compared to the range of 6.0 to 30.6 µg/g dw obtained using the conventional method. Extraction of *trans*-ferulic acid was also increased with the improved microscale method, ranging from 673.8 to 1702.7 µg/g dw, compared to 131.8 to 427.5 µg/g dw for the conventional method. The improved method also showed a reduced coefficient of variation for *p*-coumaric acid (lower than 0.15) while that for *trans*-ferulic acid showed a similar range of variance. However, most of the quantifications of *trans*-ferulic acid lay between 0.04 and 0.08, while those for the conventional method centered between 0.04 and 0.14. Therefore, the improved method achieved a higher extraction for both *p*-coumaric and *trans*-ferulic acid in their bound forms, with a reduced variation, when compared to the conventional method.

Isomers of diferulic acids were also quantified using both extraction methods, as shown in Table 2. Both extraction methods allowed the detection and quantification of 5-5 diferulic acid (5-5 DFA) and diferuloylputrescine (DFP). Significant differences were found between both extraction methods for the extraction of diferulic acids. The conventional method gave amounts of 8.6 and 6.6 µg FAE/g dw for 5-5 DFA and DFP, respectively, whereas the improved method gave values of 22.5 and 13.4 µg FAE/g dw for 5-5 DFA and DFP, respectively.

**Table 2:** Comparison of conventional macroscale and improved microscale methods for extracted bound phenolic acids founded in whole maize hybrids kernels

	*p*-Coumaric acid^a^ (µg/g dw)		*trans*-Ferulic acid^a^ (µg/g dw)		5-5 DFA^b^ (µg FAE/g dw)		DFP^b^ (µg FAE/g dw)	
	Conv	Improve	Conv	Improve	Conv	Improve	Conv	Improve
Minimum	6.0	34.4	131.8	673.8	8.6	22.5	6.6	13.4
Mean	19.4	74.2	249.7	1029.9	12.9	64.9	20.2	25.0
Maximum	30.6	138.6	427.5	1702.7	17.3	131.5	31.5	49.9
LSD	18.6	10.9	270.1	121.9	8.7	8.2	18.2	19.9
C.V.	58.9	8.9	53.8	7.2	40.5	7.6	54.7	48.1
ANOVA	***		***		***		*	

*Conv* conventional method. *Improve* improved method. *DFA* diferulic acid, *DFP* diferuloylputrescine. Significant differences between the methods were established by ANOVA (α = 0.05), *NS* no significance, * p < 0.05; ** p < 0.001; *** p < 0.001. *LSD* least significant difference (α = 0.05)

^a^Results are expressed as the average of three replicates as µg per g dry weight (µg/g dw)

^b^Results are expressed as the average of three replicates as µg FAE/g dw: µg of ferulic acid equivalents per g dw

**Fig. 4:**
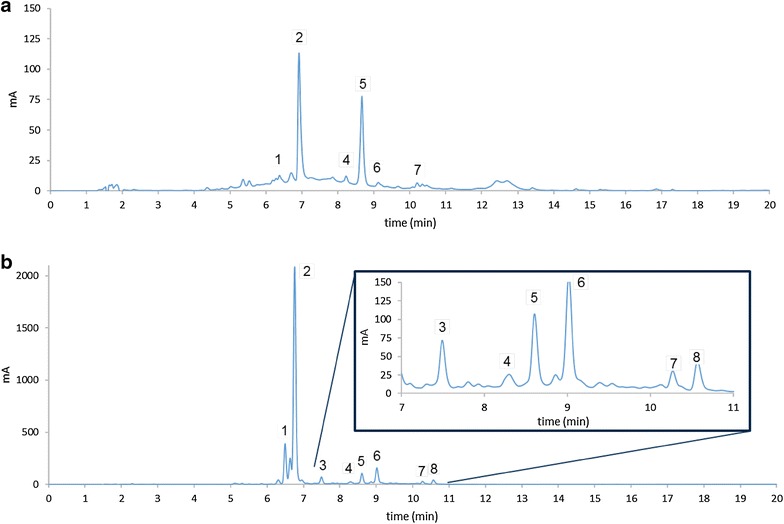
Chromatogram comparison of bound phenolic acids. **a** Conventional macroscale extraction. **b** Improved microscale extraction. Numbers inside the chromatogram denote the different peaks identified. 1. *p*-coumaric acid, 2. ferulic acid, 3. 8-5 benzofurane, 4. 5-5 diferulic acid, 5. 8-O-4 diferulic acid, 6. 5-5 diferulic acid benzofurane, 7. diferuloyl-cumaroyl putrescine. 8. diferuloylputrescine. Inset square: magnified view of the chromatogram from retention time 7 to 11 min

Figure 4 shows a comparison of the chromatograms obtained for the conventional and the improved methods. In addition to 5-5 DFA and DFP, the improved method allowed the identification and quantification of the diferulic isomers 8-5 diferulic acid, 8-0-4 diferulic acid and 5-5 DFA benzofurane. Two aromatic amides, p-coumaroyldiferuloyl putrescine and diferuloylputrescine, were also identified in some samples with a higher precision than with the conventional method. Thus, the improved microscale method achieved a better resolution and definition of diferulic acid peaks in the chromatogram, allowing their proper identification and quantification.

## Discussion

Conventional extraction of phenolic acids involves the use of different solvents that take advantage of their polarities to make a solid–liquid extraction. The major drawbacks of conventional procedures are the use of large quantities of different solvents, which generate a significant volume of toxic waste [3], and the highly time-consuming nature of the method, which makes the handling of several samples at once a challenging task.

In the present study, we compared a conventional extraction method with an improved, microscale method in yellow maize samples, and found several advantages of the improved method. It allowed a reduction in the required quantity of sample and solvents, in the extraction time, and in the amount of solvent waste generated, while increasing the number of samples that could be handled per batch. The enhanced performance of the improved method was achieved through important modifications of the conventional method, which involve solvents and solvent volumes for extraction, sample:solvent ratios, and the duration of the processes in the method. Different solvents, like alcohols, acetone, diethyl ether and ethyl acetate, have been used for phenolic acid extraction [3]. The use of only organic solvents is restricted, as extremely polar phenolic acids would not be effectively extracted. Aqueous solutions of methanol or ethanol (70–80% alcohol) are the usual solvents of choice for the first step of phenolic acid extraction; however, although methanol is more efficient at extraction, its use is limited because ethanol is considered a safer and more environmentally friendly solvent [17]. Consequently, the low solvent volume required for the improved method could make methanol an environmentally acceptable choice for extraction.

The contents of total free and bound phenolic acids, as evaluated by the conventional and improved extraction methods, gave results that were in accordance with previous reports that quantified total free and bound phenolic acids in maize [13, 18–21]. Similar amounts of total free phenolic acids were extracted using either methodology, but a greater amount of total bound phenolics was extracted with the improved method. This represents a unique and novel advantage, particularly in light of the drastic reduction in the amount of sample required. Modifications to the method for scaling down did not negatively affect the extraction of total phenolic acids. Both methodologies confirmed that the bound form represented over 75% of the total phenolic acids, in agreement with the results reported previously by Adom and Liu [2].

The phenolic acids bound to cell walls have typically been extracted using a number of different treatments. For example, alkaline or acidic hydrolysis are both possible choices. Alkaline hydrolysis breaks the ester bonds linking phenolic acids to the cell wall, while acid hydrolysis breaks glycosidic bonds and solubilizes sugars, leaving ester bonds intact [3]. The alkaline hydrolyses of cereals usually use NaOH, most commonly at a concentration of 2 M [22]. Comparisons of alkaline and acidic hydrolyses have indicated that different phenolic acid extraction profiles can be favored in each case. Alkaline hydrolysis achieves a higher extraction of total phenolic acids [23], and particularly of *trans*-ferulic acid and *p*-coumaric acid, which can suffer degradation under acidic conditions at high temperatures [24, 25].

Higher extractions with longer durations of hydrolysis have been reported [26]. However, prolonged times of alkaline hydrolysis can decrease the phenolic acid content [27]. This effect of varying hydrolysis times reveals the equilibrium required for phenolic acid extraction: longer extraction times increase the chance of higher extraction rates but also of oxidation [17]. In the case of *trans*-ferulic acid, higher hydrolysis times have been reported to decrease the recovery to 63% [2]. Therefore, finding an optimal duration for hydrolysis is important to allow higher extraction of phenolic acids without promoting degradation of these compounds. In the conventional methodology, alkaline hydrolysis is performed for 1.5 h, whereas for the improved methodology, this time was reduced to 1 h. In both methods, the temperature was set at 90 °C and the extracts were flushed with nitrogen gas to prevent oxidation. NaOH was added at 2 M in both methodologies and the solvent:sample ratio was maintained at 10:1. The improved method may establish a better equilibrium for the hydrolysis process by the adjustment of the hydrolysis duration, thereby achieving a better extraction of bound phenolic acids by a combination of increased extraction and less degradation.

The adjustment of pH after the hydrolysis treatment with the improved method had a major influence on the phenolic acid content. A precise adjustment to pH 2 must be performed to allow proper quantification of bound phenolic acids, when compared to the broader range (pH 2–3) used in the conventional method. The effect of pH on phenolic compound stability has been evaluated previously and is related to the structure of each phenol. Both *p*-coumaric acid and *trans*-ferulic acid have been identified as having a high stability to pH changes, although more complex phenolic compounds, including diferulic acids, have been found to be more susceptible to pH. The stability to pH has been associated with the number of OH groups present, their position and the number of substituents in the benzene ring [28].

A phenolic acid extraction method must consider the solvent:sample ratio as well as the number of replicate extractions, as both will affect the recovery performance [29]. In the conventional extraction, a total of 5 ethyl acetate washes are used, with a solvent:sample of 10:1. By contrast, the improved method uses only 3 ethyl acetate washes with a solvent:sample of 16:1. The increased solvent:sample ratio in the improved method could explain the higher extraction rate, as more solvent was available to interact with the matrix without having a saturation effect. The increase in phenolic acid extraction in response to an increasing solvent:sample ratio has been reported previously, although a compromise was needed to achieve a high phenolic acid extraction without an excessive increase in the solvent required, considering the high solvent costs and solvent waste generation [30]. Clearly, the conventional method has areas of opportunity, especially in terms of the solvent:sample ratio, even when a higher number of washes is employed. As already mentioned, the conventional method was less efficient at extracting the bound phenolic acids when compared to the microscale method.

One interesting result was revealed by the comparison of quantifications following the two extraction methods. No significant difference was noted between the total free phenolic acids, but differences appeared for the individual free phenolic acids (*trans*-ferulic acid and *p*-coumaric acid). By contrast, total bound phenolic acid contents, as well as individual bound phenolic acid content, were significantly higher for the improved method than for the conventional method. Therefore, in the case of free phenolic acids, the quantification of total phenolic acids apparently fails to distinguish the improvement revealed by the HPLC quantification. The Folin–Ciocalteu (F–C) assay is generally the method of choice for quantification of total phenolic acids in food samples. This colorimetric assay is based on the rapid oxidation reaction of phenols in an alkali (sodium bicarbonate). The phenolates reduce the F–C reagent, changing it into a blue pigment that is measured spectrophotometrically [31]. The F–C reaction is used extensively, but several disadvantages have been documented, most of them regarding its lack of specificity. It is an assay based on reduction, so it does not distinguish between reducing compounds other than phenolic acids that could be present in the sample. The two most recognized compounds of concern are ascorbic acid (or dehydroascorbic acid) and reducing sugars (glucose and fructose) [32]. Recently, an assay involving the use of 80 different standards showed that a broad variety of compounds, including phenols, proteins, thiols, vitamins and amino acids, show reactivity with the F–C reagent [33].

A lack of correlation has been reported previously between the F–C assay and phenolic compounds quantified by HPLC. Sánchez-Rangel et al. [32] reported quantification of the 5 major phenolic compounds in strawberry, but they found no correlation between the phenolic content quantified by HPLC and by the F–C assay, whereas a significant correlation was found between interfering substances and the F–C assay. A significant effect of interfering compounds has also been found in other matrixes, like carrot and kiwi [32]. Andjelkovic et al. [34] found a moderate correlation between HPLC and F–C assays for phenolic extracts of olive oils. In the case of maize, these effects have not been reviewed, possibly because of the low content of reducing sugars and ascorbic acid (around 0.02% [35] and 323 nmol/g fresh weight [36], respectively) and its higher content of phenolic compounds [2]. However, consideration of the presence of interferents might be important in the case of analysis of free phenolic acids. The free phenolic compounds in this study represented around 25% of the total phenolic acids, which agrees with the values reported by García-Lara and Bergvinson [18]. Note also that the extraction of free phenolic compounds is mostly based on the polarity of the compounds relative to the polarity of aqueous methanol (70%). The co-extraction of other polar molecules, like sugars, proteins and hydrophilic vitamins, during the extraction of free phenolic compounds is possible and could have significant effects on the F–C assay.

Complex phenolic acids were also identified and quantified in their bound forms. The results from both methods were similar in terms of the identity of the extracted compounds, but the higher extraction by the improved method allowed for a better identification and quantification of these compounds. Complex phenolic acids, like isomers of diferulic acid, have been studied before and are mostly associated with resistance to biotic agents in maize varieties [1, 24, 37, 38]. Phenolic acid amides, including diferuloylputrescine, have been associated with maize weevil resistance [39] and as antibiosis factors to large corn borer [40]. The importance of a correct profile generation for complex phenolic acids has been highlighted previously, as these compounds could aid in the identification of better varieties for maize improvement [1].

Another major effect of the improved method is a consistent reduction in the coefficient of variation, when compared to the conventional method. This reduction was found for the total phenolic acids as well as the individual compounds. The coefficient of variation is directly related to the standard deviation of the replicates within a sample quantification; therefore, a lower value represents a higher replicability of the extraction procedure [41]. Replicability is an essential quality for any laboratory assay, especially when a large number of samples is handled. The improved method proposed here assures high sample handling without compromising the replicability of the assay.

## Conclusions

 Conventional phenolic acid extraction takes advantage of differences in polarities to achieve a reasonable extraction rate. However, the high consumption of solvents and time make this extraction non-sustainable and a low-capacity processing method. The improved, microscale method proposed here provides an approach to overcome these issues, gaining extraction power and batch capacity with lower sample quantity, solvents and time, while also achieving a better replicability with a lower coefficient of variation than the conventional extraction. Additionally, the improved microscale method represents an efficient alternative for the management of several samples at once for phenolic extractions from maize without compromising the replicability of the assay.

## Additional files

**Additional file 1.** Determination of proximal analysis for maize varieties. *Results are expressed as the average of three replicates ± standard deviation.

**Additional file 2.** Comparison of total phenolic acids determination by conventional and improved methods. *Results are expressed as the average of three replicates as mg of gallic acid equivalent per 100 g dry weight (mg GAE/100 g dw). Min: minimum value of the data, Max: maximum value of the data. LSD: Least significant difference (α = 0.05).

**Additional file 3.** Comparison of free phenolic acids determination by conventional and improved methods. *Results are expressed as the average of three replicates as µg per g dry weight (µg/g dw). Min: minimum value of the data, Max: maximum value of the data. LSD: Least significant difference (α = 0.05).
